# Massive Demodicosis of the Eyes in a Patient with Sjögren Syndrome: A Case Report

**DOI:** 10.1007/s11686-020-00297-w

**Published:** 2020-10-31

**Authors:** Marta Ziaja-Sołtys, Magdalena Kołodziejczyk, Beata Rymgayłło-Jankowska, Dominika Wróbel-Dudzińska, Ewa Suchodoła-Ratajewicz, Dominika Szlonzak, Tomasz Żarnowski, Anna Bogucka-Kocka

**Affiliations:** 1 Chair and Department of Biology with Genetics, Medical University of Lublin, Witolda Chodźki 4 Street 4, 20-093 Lublin, Poland; 2 Department of Diagnostics and Microsurgery of Glaucoma, Medical University of Lublin, Chmielna 1 Street, 20-079 Lublin, Poland; 3 Department of General Ophthalmology, Medical University of Lublin, Chmielna 1 Street, 20-079 Lublin, Poland

**Keywords:** *Demodex *ssp., Ocular demodicosis, Sjȍgren syndrome, Therapy

## Abstract

**Purpose:**

*Demodex* mites infestation, typically asymptomatic, is a problem for patients with weakened immune systems because it often takes the form of symptomatic, massive infection. The *Demodex* mites play an important role in the occurrence of a range of eye surface diseases such as *Demodex* blepharitis, Meibomian gland dysfunctions, conjunctivitis and corneal changes. The ocular infection is closely related to the systemic invasion. Our goal was to minimize infestation and alleviate the symptoms of massive demodicosis so as to prevent further damage to the cornea.

**Methods:**

Our research note involves a 61-year old woman diagnosed with secondary Sjögren syndrome due to rheumatoid arthritis. On the background of the autoimmune disease, corneal perforation of the left eye occurred that was cured by surgery. Then during the follow-up visit the patient was found (microscopically) massively infected with *Demodex* mites and the developed symptoms were particularly severe.

**Results:**

Adequate dry eye syndrome and massive demodicosis therapy significantly reduced the number of *Demodex* mites and improved the patient’s condition.

**Conclusion:**

We would like to draw the attention of the physicians of different specialties that special care should be taken with respect to the therapy of dry eye syndrome and ocular demodicosis in patients with immunological disorders to achieve therapeutic success and avoid particularly dangerous consequences of these diseases.

*Demodex folliculorum* and *Demodex brevis,* of phylum Arthropods, are the most common ectoparasites specific for humans [1]. They inhabit different niches. *Demodex folliculorum* lives in the hair follicles, at the base of the eyelashes while *Demodex brevis* in the deeper layers of the skin in the sebaceous glands. *Demodex *spp. feeds on sebum, lymph, epithelial and gland cells. Thus both human species of *Demodex* occupy the areas with a high density of sebaceous glands especially on the face but they can be present on the whole body [2–4]. The life cycle is simple, takes place in one host organism and lasts 15–21 days. After mating in the follicle opening, the female lays about 20 eggs inside the hair follicle or sebaceous gland. The 3–4 days later, the larvae hatch, transform to the nymphs and then into the adult forms. These changes take about 7 days [5, 6]. The adult *Demodex folliculorum* can be 279–294 μm long, while the arrow-shaped egg cells reach a length of 104 μm × 41 μm. *Demodex brevis* is shorter, reaches a size of 165–208 μm, the egg cells are spindle-shaped and reach a size of 60 μm × 34 μm [7]. In healthy individuals *Demodex *spp. are normal skin microbiota. The prevalence of healthy individuals ranges from 23–100%. In the study by Wesołowska et al., the incidence of *Demodex* spp. infection among all participants (four groups: hospitalized patients, drug addicts, healthcare workers, medical students) was 41%. The highest incidence rate was recorded among hospitalized patients and the elderly [6–9]. *Demodex* mites infestation, typically asymptomatic, is a problem for patients with weakened immune systems because it often takes the form of symptomatic, massive infection [3]. It was stated that *Demodex* mites keep the balance in ocular ecosystem by cleaning the lashes from bacteria, eating them, and also prevent this niche from occupying by other species of mites. Their presence influences the immune system answer to them, regulates and buffers it [2]. In the case of *Demodex folliculorum* over-population the cylindrical dandruff, the pathognomic symptom of infestation, is observed. The mites do not have excretory organs thus they regurgitate wastes. The epithelial cells, keratin, eggs and wastes form collars around the lashes [10]. Microabrasions, mechanical blockage of the Meibomian glands orifices caused especially by *Demodex brevis* mites induce epithelial hyperplasia and reactive hyperkeratynization. Chitinous exoskeleton, excreta of the mites are foreign substances for human organism and induce granulomatous reactions [11, 12].

According to the location of the parasites, two forms of demodicosis are currently known: cutaneous and ocular. Ocular demodicosis is a chronic, inflammatory disease that is underdiagnosed because of non-specific features such as itching, redness, pain, purulence, eyelashes loss or watery eyes. It is usually connected with chronic refractory blepharistis, recurrent styes, chalazion and dry eye syndrome [13].

Sjögren’s syndrome (SS) is a chronic, progressive autoimmune disease characterized by lymphocytic infiltration of the exocrine glands in different sites of the organism but particularly lacrimal and salivary glands. Primary Sjögren syndrome is considered when arises alone, while secondary is associated with other underlying disease such as rheumatoid arthritis or systemic lupus erythhematosus [14, 15]. Lacrimal gland dysfunction causes dry eye symptoms that are not specific for Sjögren syndrome but is stated in 1/10 patients with that disease. Timely diagnosis and adequate therapy prevent ocular or systemic complications [16]. The dry eye symptoms of SS include itching, grittiness, soreness and also eye fatigue, reduced visual acuity, photosensitivity, ocular discharge, erythema. Accumulation of sticky mucus overnight makes difficult opening eyes in the morning. Sometimes there are more severe manifestations such as corneal ulceration, vascularization, opacification and very rarely, perforation [14]. It was stated that in patients with Sjögren syndrome Meibomian glands have increased number of occluded orifices, metaplasia and reduced quality of Meibomian gland secretions. Thus it is important to consider infestation with *Demodex* mites in patients who are non-responsive to Meibomian gland disease treatment [2, 17]. Reducing the number of mites is the main and more possible to achieve the goal of treating demodicosis. It is nearly impossible to eliminate the whole population. There are different options of treatment that are individually proposed to patients depending on the severity of symptoms and numbers of mites.

*Demodex* are resistant to most common antiseptic substances such as 75% alcohol, 10% povidone-iodine or erythromycin. The most effective is tea tree oil with terpinen-4-ol as antimicrobial, antifungal, antiviral, antiseptic and acaricidal active agent [2].

Representative case report: 61-year-old woman applied to the Department of Ophthalmology Medical University in Lublin in November 2016 with the corneal perforation of the left eye. She had been treated for secondary Sjögren syndrome due to rheumatoid arthritis for 27 years. The left cornea perforated 18 years ago, then the patient was treated conservatively and the perforation site healed after treatment but leaving a small corneal scar. Ophthalmological examination revealed: visual acuity of the left eye = 0.1 (without correction); visual acuity of the right eye = 0.6 (without correction). Paracentrally towards 8.30 o’clock position perforation of the cornea of the left eye was visible. Around the perforation, there was edema and light fogging of the cornea. The anterior chamber of the left eye was significantly shallow, the lens was transparent, the details of the eye's fundus were less visible. The patient was admitted to the Clinic for conservative and surgical treatment. She received: Levofloxacinum in drops every 2 h, 1% Atropine 1 × daily, artificial tears (Thealoz Duo) every 2 h. Next day after hospital admission, the surgery was performed: closing the corneal perforation of the left eye by the amniotic membrane transplantation. After the procedure, a contact lens and punctum plugs for four lacrimal puncta were put on. The patient was discharged from the Clinic in good local and general condition on the fourth day after surgery. During the third follow-up visit in March 2017 she complained of burning, discomfort, itching and redness of the eyelid margins. The ophthalmological examination revealed: visual acuity of the left eye = 0.7; visual acuity of the right eye = 0.9 c.c. + 1.0Dsph. The cornea of the left eye was smooth, the area of perforation was healed. Eyelid margins were reddened, slightly swollen. Keratin-fat cuffs suggesting a *Demodex* mites infestation were observed at the base of the eyelashes. After the on-site examination of eyelashes, the presence of numerous *Demodex* individuals were found (Fig. 1).

**Fig. 1:**
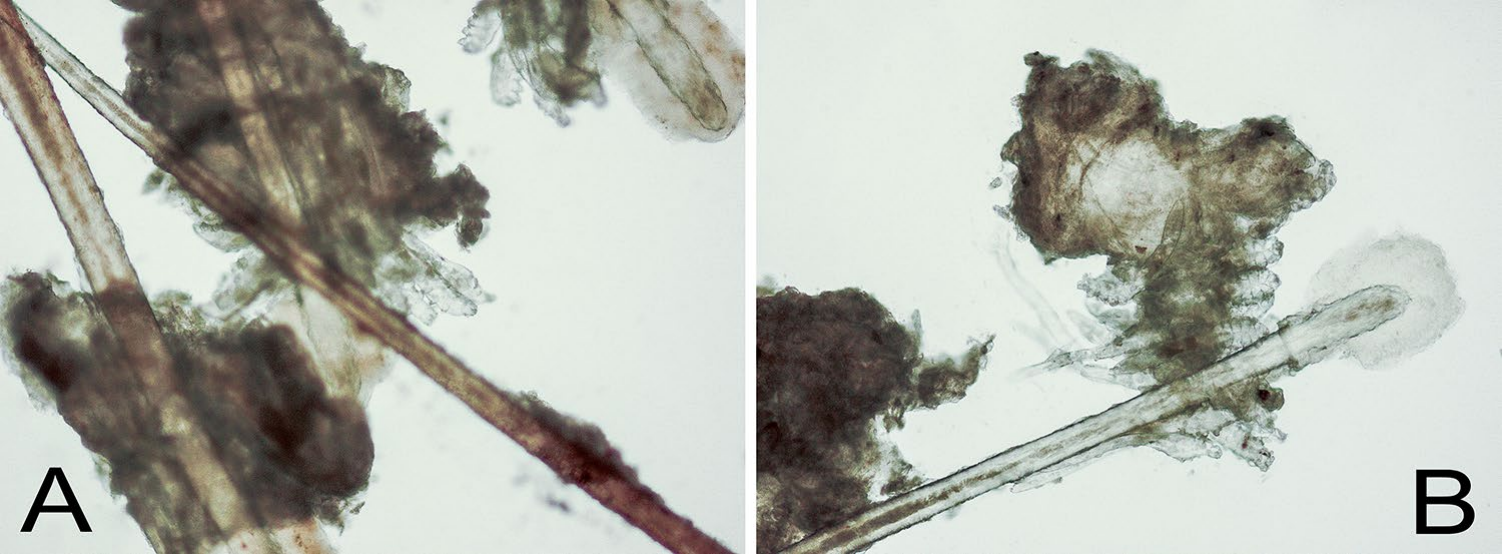
Numerous *Demodex* mites on eyelashes taken from the eyes of the patient during the visit in March 2017. Many dandruff fragments are seen. Microscopic preparation in 0.9% sodium chloride solution. Magnification 200×. **a** Right eye; **b** Left eye

Four eyelashes from each eye were taken aseptically, by means of tweezers. The eyelashes were placed on a slide, soaked with a drop of 0.9% sodium-chloride solution and studied under a light microscope using a magnification of 100 × and 200 ×. On eyelashes collected from the left and right eye of patient adult *Demodex* individuals and a few larval forms were observed. 27 *Demodex* mites were found on the eyelashes of the left eye and 30 on the eyelashes of the right eye. The *Demodex* count was recorded as a total number of adult and larval forms that were found in all four lashes taken from each eye. Our goal was to minimize infestation and alleviate the symptoms of massive demodicosis so as to prevent further damage to the cornea. The patient received anti-*Demodex* treatment: Demoxoft Lipogel (Verco) on the eyelid edges with Demoxoft liquid (Verco) and Erythromycin ointment also on the eyelid edges. During subsequent control tests in May 2017, August 2017 and in November 2017, many *Demodex* parasites on the lashes of the patient were still present (despite the maintained anti-*Demodex* treatment). Moreover, in November 2017 on consultation it was stated: V.o.d. = 1.0c.c. + 1.0Dsph; V.o.s. = 0.6; Schirmer test OP = 0 mm; OL = 1 mm. There were punctum plugs for four lacrimal puncta. The next follow-up visits took place every year in April. During the last examination subjectively, the patient felt better reported less discomfort and less noticeable burning sensation. Combined dry eye treatment based on regular (5–6 times a day) artificial tears application with four punctum plugs insertion into four lacrimal puncta together with anti-*Demodex* treatment (Demoxoft Lipogel and Demoxoft Liquid) applying to eyelid margins, used consistently from November to April), enhanced clinical condition of the eye surface and eyelid margins. The eye surface (corneal and conjunctival surface) was moist and eyelid margins were found to be more pale. Thus the patient claimed to feel better.

According to the diminished signs and symptoms of dry eye only 4 puncta plugs for lacrimal puncta were inserted. The presence of purulent and mucous secretions in the conjunctival sac from about 4 days—the patient suffered from an infection of the upper respiratory tract and rhinitis mucosa. Regarding subject: conjunctiva was paler than before. Meibomian glands—obstructed, especially on the edge of both lower eyelids. Eyelash cuffs were still present, but in the microscopic examination of eyelashes for the presence of single mature forms of *Demodex* were found (in previous studies, even a dozen of individuals in various stages of development). The tea tree oil with terpinen-4-ol (the main component of Demoxoft Lipogel and Demoxoft Liquid) used regularly on eyelid margins, is very effective in diminishing the number of *Demodex* mites [18–20]. The purulent-mucous secretion in the conjunctival sac and internal angles of both eyes as well as redness of the eye and eyelid conjunctiva may indicate bacterial conjunctivitis probably associated with upper respiratory tract infection and rhinitis (Fig. 2).

**Fig. 2:**
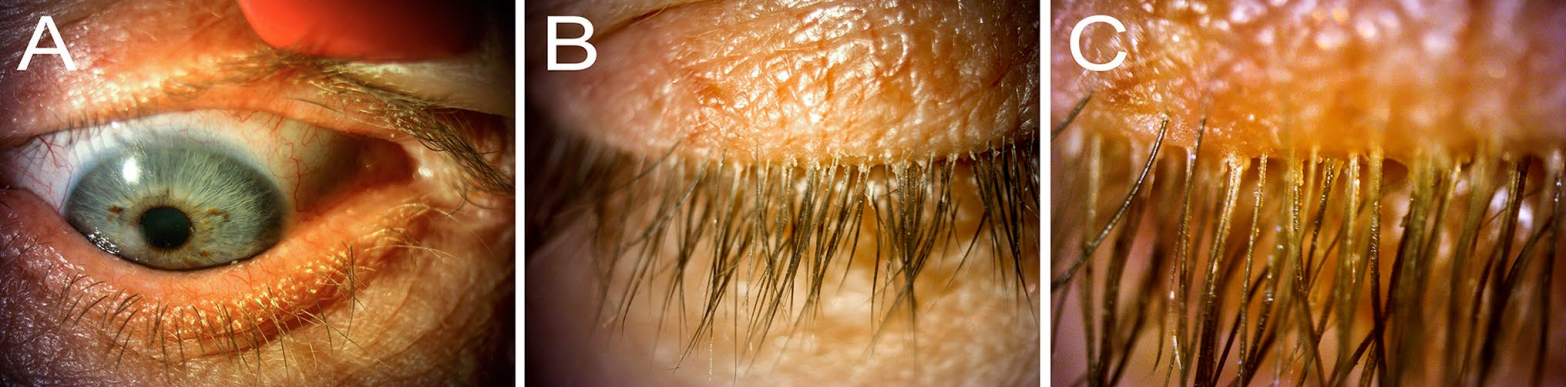
**a** Right eye. Conjunctival hyperaemia, dilated blood vessels of the upper and lower eyelid margin, clogged Meibomian glands orifices of both eyelids, collar of tissue around the base of the eyelashes of the lower eyelid. **b** Upper left eyelid. Collar of tissue around the base of the eyelashes, typical for *Demodex* infestation. **c** Collar of tissue around the base of the eyelashes, typical for *Demodex* infestation, higher magnification

The symptoms of ocular demodicosis and dry eye syndrome are similar except for the presence of cylindrical dandruff at the base of eyelashes that is characteristic of demodicosis. Meibomian gland dysfunction very often causes of dry eye syndrome. The *Demodex* mites are the most widespread but very often overlooked human parasites. It is known that *D. folliculorum* mites promote bacterial proliferation and cause inflammation of the eyelid and the conjunctiva. This enhances the production of lipases and esterases and consequently increase the viscosity and melting temperature of the meibum, reducing its secretion onto the surface of the tear film [21]. Secondary demodicosis is classified in conjunction with local or systemic immunosuppression including Sjögren’s syndrome or rheumatoid arthritis [22]. Kheirkhah et al. reported corneal changes due to demodicosis. Although *D. folliculorum* mites seem to be more prevalent than *D. brevis* they detected the last one in half of the studied cases. In addition, other authors have identified refractory and recurrent keratitis in patients with confirmed *Demodex* infestation. Interestingly these keratitis had been earlier treated as herpes ones, but therapy was not successful. Antiparasitic treatment with Cliradex (active compound terpinen-4-ol) has already given positive results after one week [18, 23].

Daily eyelid hygiene is the most important in the treatment of demodicosis. However, there is no standard treatment. There are many products on the pharmaceutical market, in the form of liquids, gels or wet wipes, containing extracts of tea tree and essential oils of sage or aloe. The toxic effect of these substances against *Demodex* was confirmed by tests. Terpinen-4-ol, an active compound isolated from tea tree oil, stimulates *Demodex* mites to come out to the surface of the skin, which facilitates the killing effect of the preparation against mites and their mechanical removal from the surface of the eyelids. Moreover, these preparations do not cause serious side effects, which enables their long-term use [19, 20, 23–26].

In the reported case, the positive effects of treatment, meaning reduction a number of observed *Demodex* mites, were obtained after the use of Demoxoft (Verco) preparations containing 4-terpinen-ol, aloe extract and Spanish sage oil. Additionally, Erythromycin ointment on the eyelids was used.

It is important that immunologists, allergists and ophthalmologists consider infestation with *Demodex* mites in patients who are non-responsive to Meibomian gland disease treatment with blepharitis or other infectious diseases of the ocular surface, unresolved with antiviral, antibacterial or antiallergic treatment. Massive demodicosis, chronic autoimmune diseases like Sjögren syndrome and rheumatoid arthritis are agents that favor dry eye syndrome development [2, 27, 28].

Timely undertaking demodicosis therapy suppresses effects of parasitic activity on the host organism that cause direct damage to the eyelid edges, acute inflammatory reaction and Meibomian glands dysfunctions. As *Demodex* are the vectors for bacteria, viruses and fungi the therapy breaks their transmission path. The case we report confirms that special care should be taken by the physicians with respect to the therapy of dry eye syndrome and ocular demodicosis in patients with Sjögren syndrome to achieve therapeutic success and avoid particularly dangerous consequences of these diseases.
